# Use of wastewater from passenger ships to assess the movement of COVID-19 and other pathogenic viruses across maritime international boundaries

**DOI:** 10.3389/fpubh.2024.1377996

**Published:** 2024-07-15

**Authors:** Davey L. Jones, Mathew Bridgman, Cameron Pellett, Andrew J. Weightman, Peter Kille, Álvaro García Delgado, Gareth Cross, Steve Cobley, Helen Howard-Jones, David R. Chadwick, Kata Farkas

**Affiliations:** ^1^School of Environmental and Natural Sciences, Bangor University, Bangor, United Kingdom; ^2^Microbiomes, Microbes and Informatics Group, School of Biosciences, Cardiff University, Cardiff, United Kingdom; ^3^Science Evidence Advice Division, Health and Social Services Group, Welsh Government, Cardiff, United Kingdom

**Keywords:** wastewater-based epidemiology, international sea travel, border crossing, passenger ferry, AMR, SARS-CoV-2 infection, import rate, public health surveillance

## Abstract

**Objective:**

The worldwide spread of SARS-CoV-2 and the resulting COVID-19 pandemic has been driven by international travel. This has led to the desire to develop surveillance approaches which can estimate the rate of import of pathogenic organisms across international borders. The aim of this study was to investigate the use of wastewater-based approaches for the surveillance of viral pathogens on commercial short-haul (3.5 h transit time) roll-on/roll-off passenger/freight ferries operating between the UK and the Republic of Ireland.

**Methods:**

Samples of toilet-derived wastewater (blackwater) were collected from two commercial ships over a 4-week period and analysed for SARS-CoV-2, influenza, enterovirus, norovirus, the faecal-marker virus crAssphage and a range of physical and chemical indicators of wastewater quality.

**Results:**

A small proportion of the wastewater samples were positive for SARS-CoV-2 (8% of the total), consistent with theoretical predictions of detection frequency (4%–15% of the total) based on the national COVID-19 Infection Survey and defecation behaviour. In addition, norovirus was detected in wastewater at low frequency. No influenza A/B viruses, enterovirus or enterovirus D68 were detected throughout the study period.

**Conclusion:**

We conclude that testing of wastewater from ships that cross international maritime boundaries may provide a cost-effective and relatively unbiased method to estimate the flow of infected individuals between countries. The approach is also readily applicable for the surveillance of other disease-causing agents.

## 1 Introduction

It is well established that effective surveillance and a timely response are essential to limit the social, health and economic impacts of rapidly spreading diseases, such as COVID-19 (1, 2). Wastewater-based epidemiology (WBE), which measures viral markers shed by infected individuals in faeces and urine, has been successfully used for surveillance of infectious diseases at a population level, including the multi-national surveillance of SARS-CoV-2 and poliovirus (3–6). Monitoring levels of SARS-CoV-2 in wastewater has, thus, provided an effective tool and early warning system to aid in public health decision-making and tracking the success of policy interventions (7–9).

Air and maritime travel represent key factors which have facilitated the global spread of SARS-CoV-2 and other viral diseases (10–12). International shipping is of particular interest due to the large volume of potentially infected passengers which may enter the country from overseas (>20 million year^−1^ in the UK) (13). The dense aggregation of people within port and dockyard areas may also facilitate infection between individuals (14, 15). Further, industrial ships and changes of crew and movement of goods in areas with multiple working personnel has the potential to cause outbreak on ships and within ports (16–20). These transmission events then have the potential to spread through the local community and to additional international ports. In a long-distance shipping context, an outbreak of SARS-CoV-2 poses serious risks to crew as they may lack the medical infrastructure or are unprepared to capably deal with issues should they arise (18, 21, 22). This also represents an issue for tourism-based cruise ships where viral (e.g., norovirus) outbreaks have regularly led to the quarantining of vessels (23, 24). A recent modelling study has also confirmed that international seaports are likely to represent a significant risk to the spread of SARS-CoV-2 (14).

Even though considerable concerns have been raised about COVID-19 transfer associated with long-haul shipping and cruise ships (7, 14), limited surveillance has been undertaken on short-haul, mass-transport passenger and freight ships. These short-haul international routes, however, may pose a greater risk for pathogen entry in comparison to longer-haul routes due to (i) the greater number of passengers involved, (ii) a lack of point-of departure/entry testing procedures, (iii) no on-board testing, (iv) less rigorous ship sanitation/cleaning, (v) the presence of pre-symptomatic passengers who travel not knowing they carry the virus, and (vi) the inability of conventional testing to capture infections (e.g., lateral flow devices) in comparison to cruise ships which rely more on PCR-based testing. Whilst wastewater testing has been deployed at international airports to evaluate the frequency of entry of infected individuals (25–27), this approach has yet to be critically tested on ships or at international ferry ports. The success of the approach, however, relies on a range of factors including the toilet behaviour of individuals, particularly on short-haul crossings, access to wastewater on the vessels and the subsequent capacity to quantify and sequence viral RNA/DNA in the samples.

Depending on the age and nature of the ship, on-board sanitation systems can vary significantly between vessels (28). In some situations, the black- and grey-water streams are kept separate, which is ideal for WBE, whilst in others they are mixed, leading to dilution of the viral signal. In other cases, sewage is collected on the boat and then delivered to a port reception facility for subsequent treatment (29). Access to sewage collection tanks may also be problematic on some vessels whilst addition of different sanitation agents (e.g., disinfectants) may cause issues in viral recovery. Conversely, the presence of low-water use vacuum toilets can be expected to result in more concentrated sewage in comparison to on-shore municipal sewage which may aid viral detection (30).

Due to the current paucity of information, the aim of this pilot study was to critically evaluate whether wastewater-based surveillance on short-haul international passenger/freight ships is viable for monitoring the frequency of entry of SARS-CoV-2, alongside other pathogens (e.g., norovirus, influenza-A and B, enterovirus). The study focused on the main UK to Republic of Ireland passenger route, monitoring wastewater on two of the main commercial vessels over a one-month period. The work focused on the practicality, economic viability and usefulness of the approach within the framework of a potential national border surveillance programme for pathogens of public health concern.

## 2 Materials and methods

### 2.1 Sampling locations

The project was based on the maritime route between the Holyhead Ferry Terminal located in Gwynedd, Wales, United Kingdom (53°18′58.47″N, 04°37′24.47″W) and Dublin Port located in Dublin, Ireland (53°20′57.13″N, 06°11′50.70″W). The route represents the main maritime freight and passenger link between the UK and Ireland with an estimated 1.9 million passengers per year and *ca.* 450,360 cargo truck transfers (31). The route is *ca.* 80 km from port-to-port and takes *ca.* 3 h 15 min per crossing and is serviced by several commercial companies (Supplementary Figure S1). This study focused on two superferries, namely the Stena Estrid and the Stena Adventurer (Stena AB, Gothenburg, Sweden; Supplementary Figure S2).

The Stena Estrid was built in 2019 by AVIC Weihai, Shandong Province, China and is classified as an ‘E-Flexer’ passenger roll-on/roll-off cargo (Ro-Pax) ferry. It has a capacity of 1,000 passengers, 120 cars and 210 freight vehicles. The Stena Adventurer was built in 2003 by Hyundai Heavy Industries, South Korea and is also a Ro-Pax ferry with a capacity of 1,500 passengers and 500 cars and freight vehicles.

The ships possess different wastewater management systems and thus the sampling strategy varied slightly between ships. The Stena Estrid wastewater system is separated into 2 initial chambers: (i) blackwater (raw sewage from toilets), (ii) greywater (water from sinks, showers, and kitchen appliances). These are then combined in a mixing chamber and then transferred to a screening tank to remove large non-biodegradable solids. Once mixing had occurred, wastewater is transferred to an Evac Membrane Bioreactor treatment module (Evac Oy, Espoo, Finland). Post aerobic treatment, clean water is then discharged at sea whilst the solid waste becomes a dry powder that is offloaded at shore for disposal. Samples were initially planned to be taken from blackwater chamber, however, due to access/system constraints, samples had to be taken from the screening tank, but prior to any treatment occurring (Supplementary Figure S3). The Adventurer has an older wastewater system containing of 3 chambers involving maceration (soaking), chopping and mixing. After being mixed, the wastewater is moved to a similar treatment plant to the Stena Estrid where it is aerobically treated and filtered in a containment tank where it is stored until it reaches port and then taken to a wastewater treatment plant. Samples on the Adventurer were taken prior to the anaerobic treatment stage.

### 2.2 Sample collection

Wastewater sampling was undertaken on Sunday, Tuesday, and Thursday on each ship from the 27th January 2022 to the 23rd February 2022. On each day, 4 independent samples were taken representing the 4 single leg journeys between Holyhead and Dublin each day (Supplementary Table S1). The samples (500 mL) were collected by the engineering crew, placed within polycarbonate bottles and refrigerated at 4°C on the ship prior to collection from the port each day. Samples were collected directly from Holyhead port and then transported to the laboratory (40 km distance) in a refrigerated box where the samples were then stored at 4°C and analysed within 24 h of collection. Basic training was provided to the ship’s staff for sample collection.

### 2.3 Viral concentration, nucleic acid extraction, and quantification

Viral recovery and purification were undertaken according to the polyethylene glycol (PEG)-salt precipitation of Farkas et al. (32) and Kevill et al. (33). This method was chosen as it is used in the Welsh Government national wastewater COVID-19 surveillance programme. Briefly, 200 mL of each wastewater sample was placed in a sterile polypropylene centrifuge bottle and centrifuged (10,000 g, 10 min, 4°C) to remove suspended solids. 150 mL of the clarified supernatant was then transferred to a sterile polypropylene centrifuge bottle, the pH adjusted to 7.0–7.5 and 50 mL of a PEG-8000-NaCl solution added to reach a final PEG-8000 concentration of 10% and NaCl content of 2%. An aliquot of dsRNA *Pseudomonas* phage Phi6 was then added to the sample as an extraction control and the samples incubated at 4°C overnight. Post-incubation, the samples were centrifuged (10,000 g, 30 min, 4°C). The supernatant was then discarded and the pellet resuspended in 850 μL of Nuclisens lysis buffer (BioMerieux, France). The viral RNA and DNA from the resuspended pellet was then extracted using a KingFisher 96 Flex automated purification system (Thermo Scientific, Waltham, United States) using NucliSens extraction reagents (BioMérieux, France) as described elsewhere (33). The final volume of the RNA/DNA eluent was 100 μL.

One-step RT-qPCR for the SARS-CoV-2 N1 gene region and Phi6 targets was performed using an TaqMan™ Fast Virus 1-Step Master Mix (Applied Biosystems Inc., United States), on a Quant Studio Flex 6 (Applied Biosystems Inc., United States) using previously published primers and probes (34, 35) (Supplementary Table S2). The mastermix contained 10 pmol of the forward, 20 pmol of the reverse primers and 5 pmol probe, 16 nmol MgSO_4_, 1 μg bovine serum albumin (BSA), molecular grade water and 4 μL sample/standard/control in 20 μL reaction mix. RT-qPCR settings were: Hold step 50°C 30 min for reverse transcription, 95°C 20 s for reverse transcriptase inactivation, followed by 45 amplification cycles of 95°C 13 s, 60°C 45 s.

Multiplex RT-qPCR assays were used for the detection of influenza A/B viruses (flu A and flu B) and for Enteroviruses (EV), enterovirus D68 (EV-D68) and norovirus GII (NoVGII) using previously published primers and probes (36–38) (Supplementary Table S2). The same reaction conditions as for SARS-CoV-2 quantification were used except that the mixture contained no added MgSO_4_.

For crAssphage an established assay using the QuantiFast qPCR mix was used (33) with 2 μL sample added to 20 μL reaction mix.

All samples were run in duplicate, against a dilution series (1–10^5^ copies μl^−1^ per reaction) of in house developed ssRNA standards for SARS-CoV-2 and phi6 (33), commercial ssRNA standards for flu A/B and EV-D68 (Twist Bioscience, United States) or plasmid DNA for NoVGII and crAssphage (39, 40). PCR no template controls (molecular-grade water) determined the absence of contamination during the PCR set-up.

### 2.4 SARS-CoV-2 sequencing

Selected RNA extracts were further purified with Mag-Bind® TotalPure NGS beads (Omega Bio-Tek) to remove potential inhibitors prior to reverse transcription into cDNA with LunaScript® RT SuperMix (NEB) prior to SARS-CoV-2 amplification and sample indexing using EasySeq™ SARS-CoV-2 kit (Nimagen). The protocol used has been customised previously for use with wastewater (41). Amplified products were quantified and quality controlled using Agilent TapeStation. Libraries were sequenced on an Illumina MiniSeq benchtop sequencer, producing 2 × 150-bp paired-end reads. Raw reads were processed using the ncov2019-artic-nf Nextflow pipeline (42). Briefly, reads were trimmed using Cutadapt v1.18 (43) and Nimagen V4 primer sequences were removed using iVar v1.3. Cleaned reads were aligned to the SARS-CoV-2 reference genome Wuhan-Hu-1 (MN908947.3) (44) using the Burrow-Wheeler Aligner (BWA) (45) and *ca.* 400,000 reads mapped per sample. Lineage abundances were then determined using the processed sequences using depth-weighted de-mixing of SNV frequency at each position in the genome using Freyja pipeline (46, 47).

### 2.5 Wastewater physical and chemical analysis

The samples were analysed for a range of key physicochemical markers of wastewater quality including pH, turbidity, electrical conductivity (EC), ammonium and orthophosphate (9). Turbidity was assessed using an Orion AQUAfast AQ3010 turbidity metre (Thermo Scientific, Waltham, MA, United States) whilst EC was measured using a Jenway 4,520 conductivity metre and pH with a Hanna 209 pH probe (Hanna Instruments Ltd., Leighton Buzzard, United Kingdom). For NH_4_^+^ and P analysis, the samples were first centrifuged (24,000 g, 5 min) to remove suspended solids. The supernatant was then retained for subsequent analysis. Inorganic P was measured colorimetrically using the molybdate blue reagent according to Murphy and Riley (48) whilst NH_4_^+^ was determined colorimetrically using the salicylate procedure of (49) using a SpectroStar Nano microplate reader.

### 2.6 Data analysis

The qPCR quality control was carried out with QuantStudio real-time PCR software v1.7 (Applied Biosystems, Inc., United States). The standard curve slope, efficiency and R^2^ met the requirements described in Bustin et al. (50). The qRT-PCR data was converted to gc l^−1^ wastewater for statistical analysis. The assay limit of detection (LOD) and limit of quantification (LOQ) were tested using 10 replicates of low dilutions of genomic RNA for the RNA virus targets and plasmid DNA for crAssphage (40). The LOD was defined as the minimum concentration whereby 10 replicates all return positive results and the LOQ was the lowest concentration where the coefficient of variation was lower than 0.25 (Supplementary Table S2). As such, quantities can be detected below this limit but are susceptible to false negatives. For comparison, the wastewater composition from the ships was directly compared with that collected as part of the national surveillance programme undertaken in Wales. The latter involved the analysis of wastewater collected from 44 centralised wastewater treatment plants across Wales 5 days a week.

To theoretically estimate the number of a- and pre-symptomatic passengers who were travelling on the transnational shipping route (i.e., import rate, *IR*) we used the following equation:

(1) IR=PN×PP×ACR×FSR×TU

where *PN* is the total number of passengers sampled during the wastewater testing campaign (*n* = 6,942), *PP* is the prevalence of COVID-19 in the population (3.1%–4.1% of the population), *ACR* is the amount of COVID-19 cases that are pre- or a-symptomatic (20%–30% of the total), *FSR* is the shedding frequency of SARS-CoV-2 in faeces (40%–60% of cases), and *TU* is the likelihood that passengers will use a toilet whilst on board the ship (13%). It was assumed that symptomatic passengers would not be travelling due to government travel restrictions in place when the study was undertaken.

## 3 Results

### 3.1 Prevalence of COVID-19 cases during the survey period

Wastewater sampling commenced towards the end of the third main COVID-19 wave in the UK which was associated with the emergence of the omicron variant of SARS-CoV-2. During this sampling period 0.1% to 0.2% of the UK and Irish population tested positive for SARS-CoV-2 (51). Overall, the patterns in COVID-19 cases were similar between countries. Based on the results of the COVID-19 Infection Survey (CIS), which is less prone to self-reporting bias, it is likely that the true prevalence of COVID-19 in the UK and Ireland populations ranged from 3.1% to 4.5% during the study period (51–54). At the time that the wastewater monitoring was undertaken, the wearing of face coverings was still mandatory and recommendations were in place for individuals not to travel if they had tested positive for SARS-CoV-2. Stena line staff were also asked to self-isolate if they tested positive for COVID-19. At the time of the study, passenger locator forms were not required to enter the UK and no quarantining procedures were in place. Due to the COVID-19 pandemic, the number of passengers per journey was lower than normal with each journey having an average of 154 passengers (range 38 to 612) on the Stena Estrid and 169 on the Stena Adventurer (range 28 to 775). Of these, 74% were crossing with cars or as foot passengers and 26% as commercial freight drivers. There were no differences in the passenger:freight ratio between the two ships.

### 3.2 SARS-CoV-2 and other viruses in ferry wastewater

**Figure 1:**
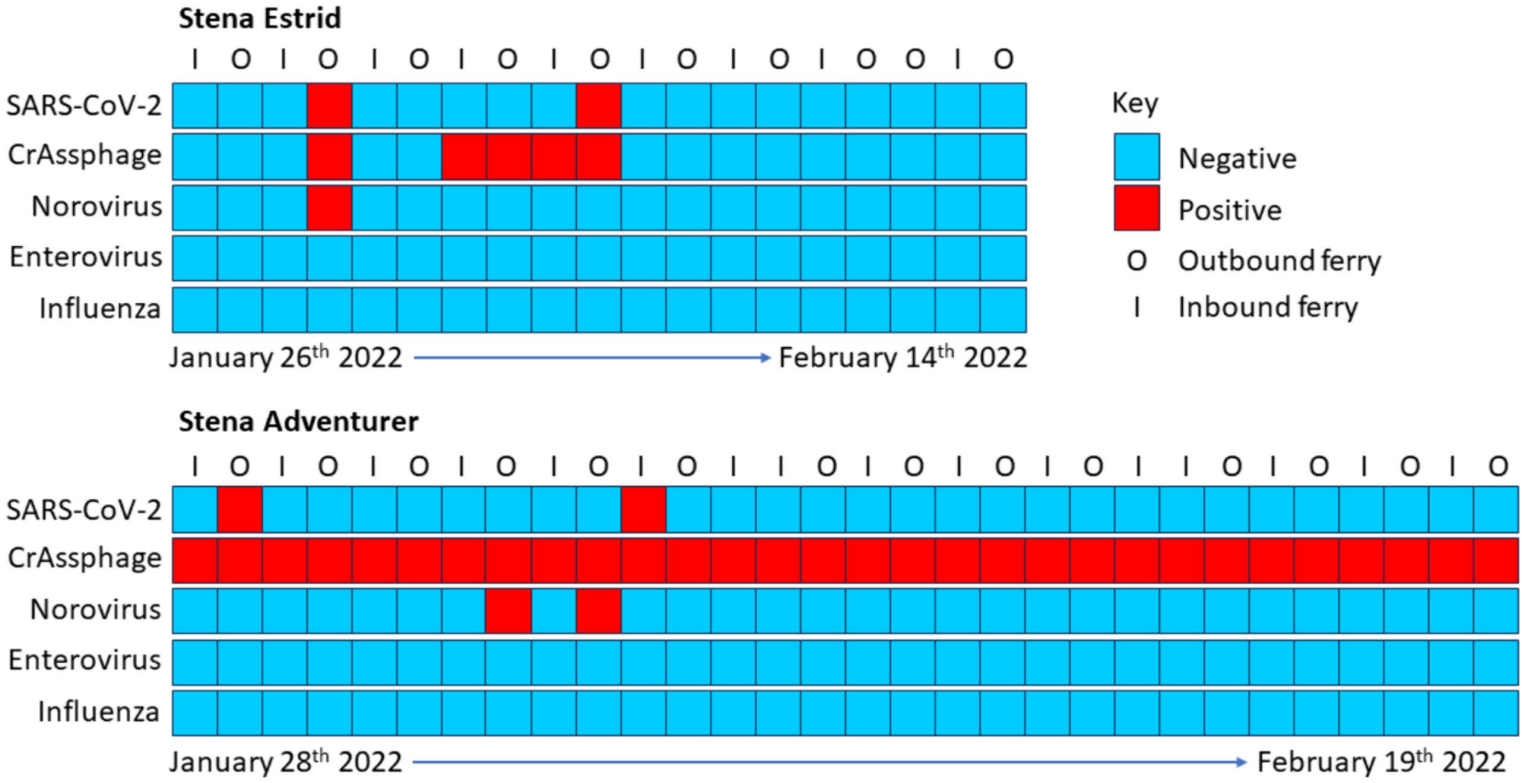
Viral detection and quantification in wastewater collected from two international short-haul ships (Stena Estrid and Stena Adventurer) taking passengers and commercial loads to and from Ireland (Dublin) and Wales (Holyhead). The outbound route is Holyhead to Dublin and the inbound route is Dublin to Holyhead. SARS-CoV-2 used the N1 gene target. The faecal-marker virus crAssphage was used as an indicator of faecal matter being present. Norovirus represents genogroup II and Influenza represents both influenza A and B. Each square represents an individual ferry crossing between Ireland and the Wales.

SARS-CoV-2 was detected in four samples during the survey period (8.1% of the total samples, *n* = 49, Figure 1). Of the positive wastewater samples, the maximum concentration detected was 9.2 × 10^5^ gc l^−1^. Of the other human pathogenic viruses tested in the wastewater samples, only NoV GII was detected, albeit at a lower frequency (6.1% of the total samples) with a maximum concentration of 1.3 × 10^6^ gc l^−1^. Neither, enterovirus, enterovirus D68 or influenza A or B were detected in the samples. The faecal marker crAssphage was detected in all samples from the Stena Adventurer, however, recovery of crAssphage from the Stena Estrid was much lower (26% of the total samples). The mean recovery of crAssphage was 1.9 × 10^6^ gc l^−1^ on the Stena Adventurer which was lower than from the Stena Adventurer when samples tested positive (2.1 × 10^7^ gc l^−1^, *p* = 0.002). Overall, the levels of crAssphage were lower than those reported in the national urban wastewater surveillance programme (mean 1.0 × 10^9^ ± 3.0 × 10^7^ gc l^−1^; *p* < 0.001).

### 3.3 SARS-CoV-2sequencing

The samples that tested positive for the SARS-CoV-2 N1 gene region by RT-qPCR were subsequently sequenced. Sequence was acquired for 600–362,000 reads of which between 60% and 82% of the mapped to the viral genome. Although this yielded an average coverage > 1,500, sequences mapped to very restricted regions of the virus and therefore provided incomplete coverage for all samples. Overall, the percentage genome covered ranged from 18% to 35%. Consequently, we were able to ascribe one sample to the SARS-CoV-2 omicron variant, however, the other three positive samples remained unascribed. The success of sequencing appeared directly related to the amount of SARS-CoV-2 recovered in the sample.

### 3.4 Wastewater chemistry

**Figure 2:**
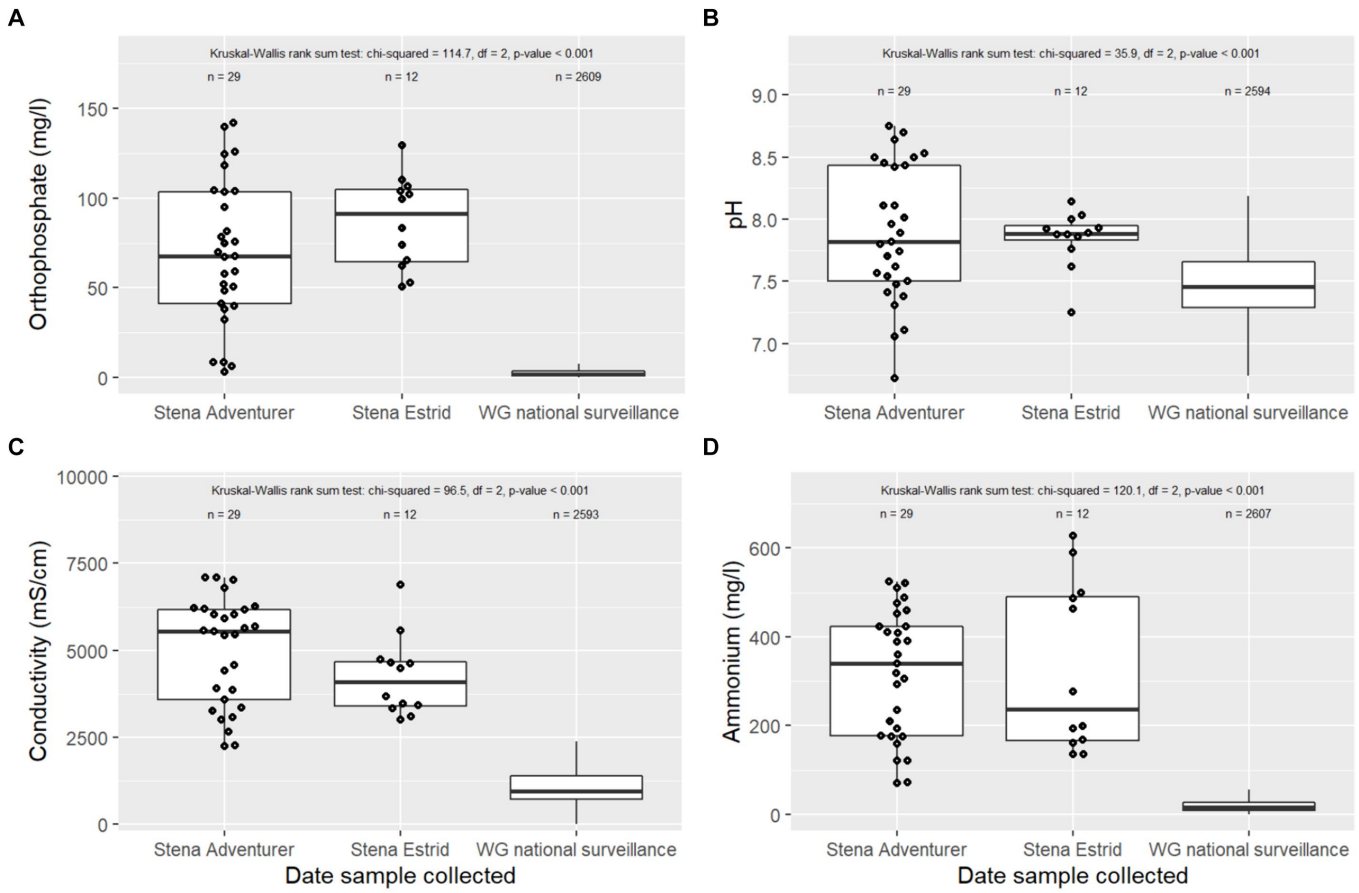
Chemical indicators of wastewater quality from two international short-haul ships (Stena Adventurer and Stena Estrid) on the international Ireland (Dublin) to Wales (Holyhead) route. **(A)** orthophosphate, **(B)** pH, **(C)** electrical conductivity, and **(D)** ammonium. For comparison, we present results for influent wastewater from 44 sites collected as part of the Welsh national COVID-19 wastewater surveillance network. The 25th, 50th, and 75th percentile ranges are depicted by the box, excluding outliers greater or lesser than 1.5 × IQR depicted by the whiskers.

The average orthophosphate concentration of wastewater on the two Stena ships (mean ± SEM, 211 ± 57 mg l^−1^) was considerably higher than samples collected during the Welsh government national surveillance project (2.6 ± 0.1 mg l^−1^; Figure 2A). Likewise, we found the median ammonium concentration of wastewater on the ships (320 ± 25 mg N l^−1^) to be much higher than the national surveillance median (16 ± 1 mg N l^−1^; Figure 2D). Further, the turbidity of the ships’ wastewater samples (1,172 ± 122 NTU) was higher that reported for urban wastewater in the national surveillance programme (90 ± 5 NTU). Similarly, the electrical conductivity and pH of the ships’ wastewater (4.7 ± 0.2 mS cm^−1^ and 7.9 ± 0.12, respectively) were also different to the national surveillance programme samples (0.9 ± 0.1 mS cm^−1^ and 7.5 ± 0.02, Figures 2B,C). None of the wastewater characteristics had significant correlations with passenger data (*p* > 0.05; data not presented).

## 4 Discussion

### 4.1 Potential of ship wastewater to capture the presence of infected individuals

Wastewater can potentially provide a non-invasive, ethically compliant and relatively unbiased way to evaluate levels of infection within a cohort of individuals all connected to a common sanitary system (5). To our knowledge, this is the first use of ship-based wastewater-based surveillance to assess the potential transfer of viral pathogens across an international maritime boundary. Our results provided clear evidence that, albeit infrequent, infected individuals were crossing between the UK and Ireland during the third COVID-19 wave when guidance was still in place to prevent travel for infected individuals. Whilst wastewater analysis has previously been undertaken on ships, this has largely been from the perspective of discharging pollutants into marine waters rather than assessing the presence of infected individuals on a vessel (55–58). Further, most of this work has focused on organic pollutants (e.g., antibiotics) and faecal-derived bacteria rather than on viruses (30, 55, 59). A single study from a cruise ship carrying passengers infected with SARS-CoV-2 showed previously that viral RNA could be isolated from the ship’s wastewater (60), providing the first evidence that wastewater can be used for on-board pathogen surveillance. However, long-haul cruise ships hold an isolated population where it can be guaranteed that all individuals will use the toilet facilities. Further, cruise ships are notorious for large viral outbreaks due to the close confinement of passengers over long periods of time (e.g., norovirus, influenza) (61–63). This suggests that viral titers in wastewater from cruise ships are likely to be very high and may also prove useful as a temporal indicator of outbreak progression.

In the case of short- and medium-haul passenger ferries (journey time < 6 h in duration), the frequency that individuals defecate remains unknown; however, it is expected that this will be very low in comparison to cruise ships. The continual changing of passengers (4 times daily in this study), is also likely to lead to more temporally stochastic results with lower viral titers (due to a higher urination-to-defecation ratio). Unlike cruise ships, in the context of short-haul shipping routes, it is the frequency of detection that is most important rather than the quantitative analysis of the amount of viral RNA present.

### 4.2 Theoretical vs. actual measured incidence of infected individuals

The success of viral surveillance using wastewater relies largely on faecal shedding and to a lesser extent vomiting and sputum, whilst very few pathogenic viruses are shed in urine (64). Previous studies have indicated that enteric and respiratory viruses are shed in faeces whether individuals are asymptomatic or symptomatic (65–67). The frequency, duration and amount of faecal shedding, however, can vary significantly between viruses, point in the infection cycle and on the nature of the individual (e.g., age, immune status etc.). Here we take a first principles approach to estimating the likely number of passengers infected with SARS-CoV-2 who can theoretically be captured using a wastewater-based approach. Although information exists for defecation frequency on long-haul ships, which suggests that most people defecate less often than on land (68), no quantitative information exists for defecation frequency on short-haul passenger ferries. Based on estimates of likely frequency of on-board defecation on short-haul flights (<13%; <3 h in duration) (69), we use this to estimate the chances of capturing infected individuals on short-haul passenger ships. Based on the total number of passengers sampled during the study period (*ca.* 6,942), a population-level COVID-19 prevalence rate of 3.1%–4.5% (51, 52), an asymptomatic carriage rate of the omicron variant of 20%–30% (70, 71) and a SARS-CoV-2 faecal shedding rate of 40%–60% (66), we estimate that theoretically the number of infected passengers would range from 2.2 to 7.3 (Equation 1). The number of actual samples which tested positive for SARS-CoV-2 (*n* = 4) directly falls within this range. One assumption we have made is that symptomatic people did not travel based on government guidance at the time of the study and that diarrhoea is not a primary symptom of omicron infections, the dominant variant in circulation at the time (72).

### 4.3 Use of wastewater for the surveillance of other viral pathogens

Although the main premise of this study was to evaluate the use of wastewater for COVID-19 border surveillance, we showed that the approach can also be used to evaluate the prevalence and movement of other viruses and is likely suitable for other disease-causing agents (e.g., anti-microbial resistant bacteria, protozoa). Here we also detected the RNA of norovirus in wastewater on several occasions. Indeed, wastewater may be better for the surveillance of enteric viruses as the frequency and volume of defecation is much greater (e.g., diarrhoea), viral shedding rates occurs in all infected individuals and the rates of shedding are much greater (66). Enteric viruses also represent the leading cause of illness amongst returning travellers seeking medical care (73). Previous estimates of trans-border movement of norovirus have relied on the analysis of serum or stool samples, largely provided voluntarily from symptomatic individuals (73–76). In combination with genotyping (to assess unique lineages), wastewater could provide an unbiased assessment of norovirus entry into the country, particularly as *ca.* 10% of infections are asymptomatic and shed at similar rates to symptomatic individuals (77). The levels of norovirus circulating in the population at the time of the study were atypically low due to the COVID-19 pandemic (78, 79) suggesting that more cases may be detected post-pandemic. Similarly, the prevalence of influenza A/B and enterovirus were also unseasonably low in the population at the time of sampling, due to the knock-on effect of non-pharmaceutical interventions for COVID-19 control (80, 81). It would therefore be useful to undertake a repeat survey under more representative circumstances to evaluate the use of wastewater for catching these viruses.

### 4.4 Limitations of using a wastewater-based approach for pathogen surveillance on ships

Whilst wastewater analysis proved successful at showing the passage of infected individuals between the UK and Ireland, the approach has some limitations and areas for refinement as follows: (i) *Sampling approach*: For logistical reasons, we relied on taking several manual spot measurements per journey rather than deploying an automated time-integrated composite sampler. Although some mixing of the wastewater will occur within the sanitary network, it is known that a grab/spot-sampling approach does not provide the most reliable estimate of viral load, particularly for near-source testing (82). The design of a refrigerated autosampler that can retrieve a wastewater sample from a pressurised sanitary network at regular intervals (*ca.* every 10 min) would therefore be useful. Further, passive sampling approaches may be appropriate to capture time integrated information without having to rely on complex autosamplers (83); (ii) *Independent validation*: To better validate the wastewater approach, it would be useful to take nasopharyngeal swabs from a representative sample of individuals to confirm the presence/absence of SARS-CoV-2 and influenza (63). Due to ethical and social considerations, validation for enteroviruses may be more problematic; (iii) *Defecation behaviour*: As the approach relies on shedding viruses in faeces, it would be useful to gain insight into the toilet habits of individuals and whether these are influenced by demographic factors (e.g., age, gender, nationality), passenger type (e.g., commercial truck drivers vs. tourists, journey details), timing (e.g., day vs. night voyages), season (e.g., tourist season vs. off-peak) and the health status (e.g., evidence of respiratory or gastrointestinal symptoms). This could be achieved by eliciting a passenger questionnaire on departure from the port. Alternatively, the number of individuals defecating on the boat could be assessed by the unique lineages of phages present in the human gut (e.g., crAssphage) (84). The toilet use by crew should also be a factor that needs to be considered in this analysis; (iv) *Wastewater transit time*: Although the samples were taken on a daily basis, the residence time of the wastewater in the sanitary network (e.g., holding tanks) (30), and therefore the potential loss of viral RNA/DNA remains unknown. Based on previous studies on marine wastewater discharges, we therefore recommend the deployment of a rhodamine tracer for mapping residence time (85); (v) *Origin of infection*: Due to the uncertainty in wastewater transit time, we were unable to determine with certainty whether the wastewater collected was from the UK-Ireland or Ireland-UK leg of the journey (or a mixture of both). The geographical origin of SARS-CoV-2 or norovirus in our samples could therefore not be determined with certainty. More complete genetic sequencing of the viral strains and mapping the lineages to national databases will clearly aid in this. Due to the high number of clinical samples being sequenced for SARS-CoV-2 this should be effective; (vi) *Viral recovery*: A preliminary investigation in a small number of samples showed that variations on the PEG-salt based method used here may give better viral recoveries. Given the concentrated nature and high urea content of ship blackwater (86), it is likely that improved methods for viral recovery and removal of PCR inhibitors is still needed. This is evidenced by the inability to recover crAssphage from some samples, despite its high abundance in human faeces from industrialised countries (87). Given the high solids content in the wastewater, it may also be desirable to evaluate the partitioning of viruses between the solid and liquid fraction so that the most enriched fraction can be targeted for further surveillance activities; (vii) *Other shipping routes*: This study targeted short-haul journeys, however, adopting a similar approach on longer maritime crossings would provide additional value and may be less affected by some of the limitations highlighted above. For example, the UK-Spain passenger ferry (Portsmouth-Santander) has a duration of 28.5 h, whilst the UK-Belgium route (Hull-Zeebrugge) takes 13.5 h and the UK-Norway (Harwich-to-Esbjerg) passage takes 18 h.

## 5 Conclusion

This study has successfully demonstrated that ship blackwater can be used to isolate and identify viruses of public health concern. Further, the frequency of detection was consistent with theoretical estimates based on known infection rates within the population. Although some refinement of the methodology is still required, we conclude that this wastewater-based approach can be readily expanded to a wide range of faecal-borne pathogens. In combination, the methodology presented here provides a non-invasive way to assessing the frequency of pathogen transfer across international maritime boundaries and thus the contribution of maritime traffic to the global spread of disease.

## Data availability statement

The data presented in the study are deposited in the Zenodo repository, accession number https://doi.org/10.5281/zenodo.1267108.

## Author contributions

DJ: Data curation, Formal analysis, Funding acquisition, Investigation, Methodology, Project administration, Resources, Supervision, Writing – original draft, Writing – review & editing. MB: Investigation, Writing – review & editing. CP: Data curation, Formal analysis, Software, Writing – review & editing. AW: Conceptualization, Funding acquisition, Project administration, Supervision, Writing – review & editing. PK: Data curation, Formal analysis, Funding acquisition, Methodology, Supervision, Writing – review & editing. ÁGD: Data curation, Investigation, Methodology, Writing – review & editing. GC: Conceptualization, Funding acquisition, Validation, Writing – review & editing. SC: Conceptualization, Funding acquisition, Resources, Writing – review & editing. HHJ: Investigation, Methodology, Resources, Writing – review & editing. DC: Funding acquisition, Resources, Supervision, Writing – review & editing. KF: Data curation, Formal analysis, Investigation, Methodology, Validation, Writing – review & editing.
