# Supplementary material for: Use of wastewater from passenger ships to assess the movement of COVID-19 and other pathogenic viruses across maritime international boundaries
Source: Front Public Health. 2024 Jul 15;12:1377996. doi: 10.3389/fpubh.2024.1377996 (PMC11284076; doi:10.3389/fpubh.2024.1377996)
Supplement: Supplementary file 1 [file Data_Sheet_1.pdf]

Supplementary Material

Supplementary Figure 1. Map showing all the main transnational passenger ferry routes between the UK and Europe. The one studied here was the Holyhead-Dublin route.

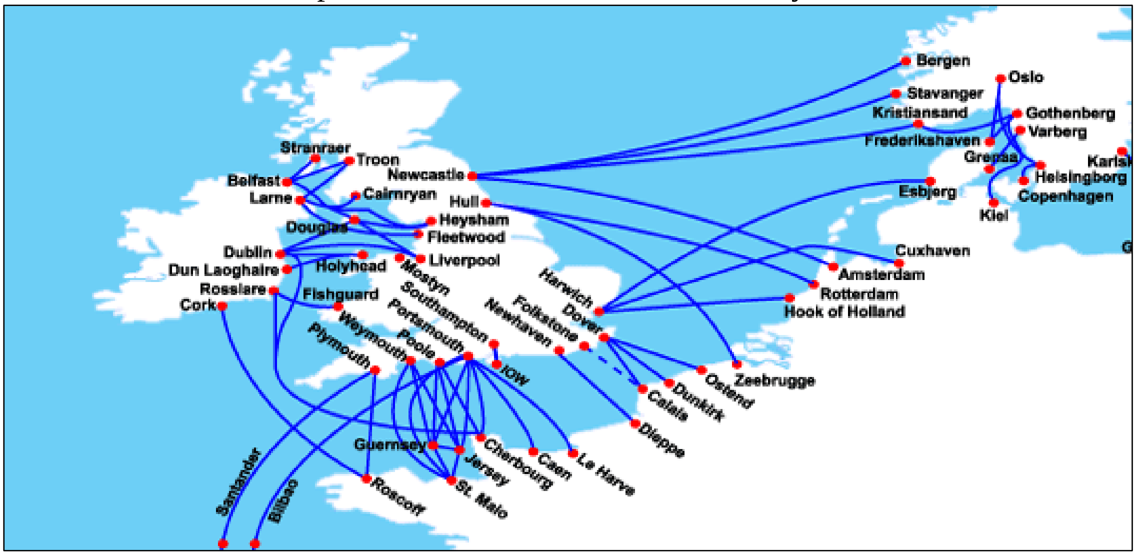

Supplementary Figure 2. Images of the Stena Adventurer (upper image) and Stena Estrid (lower image) and the used as the pilots for wastewater testing on trans-national ferries.

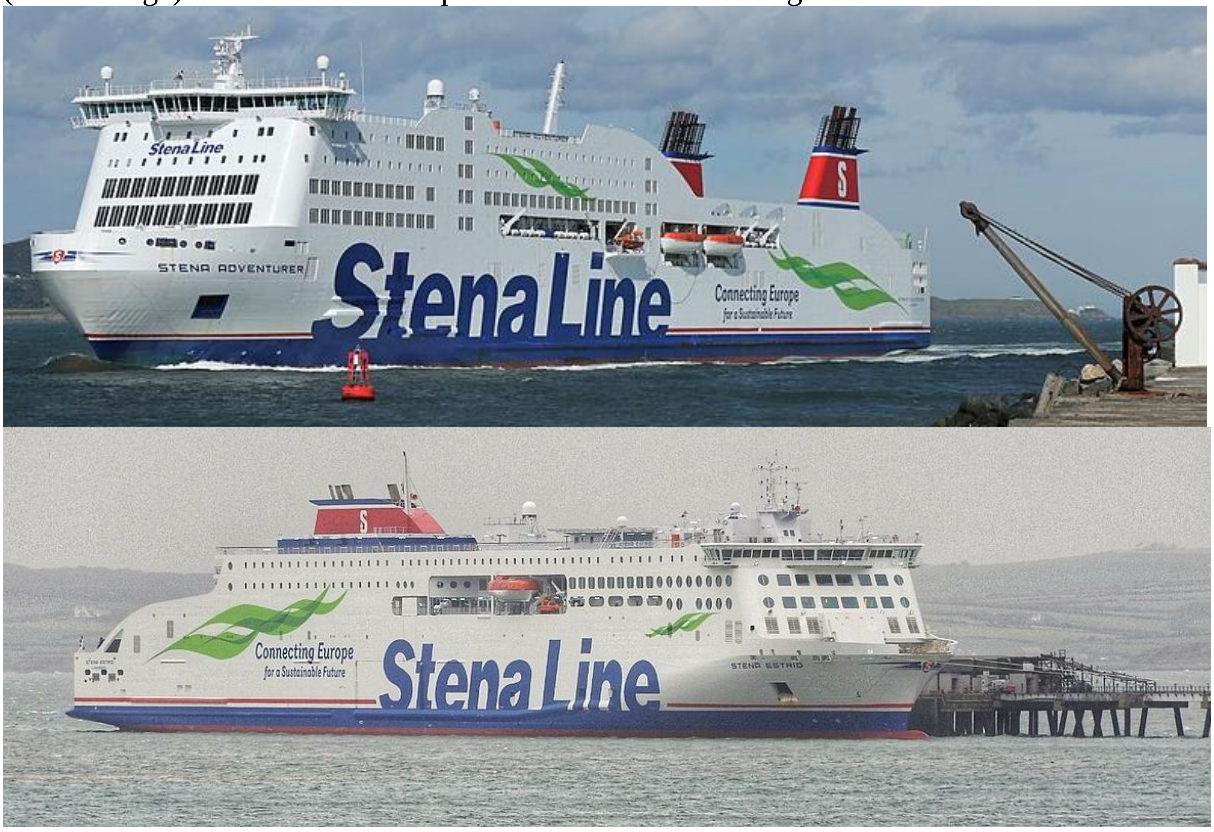

We thank Hefin Owen and CitySwift for the images.

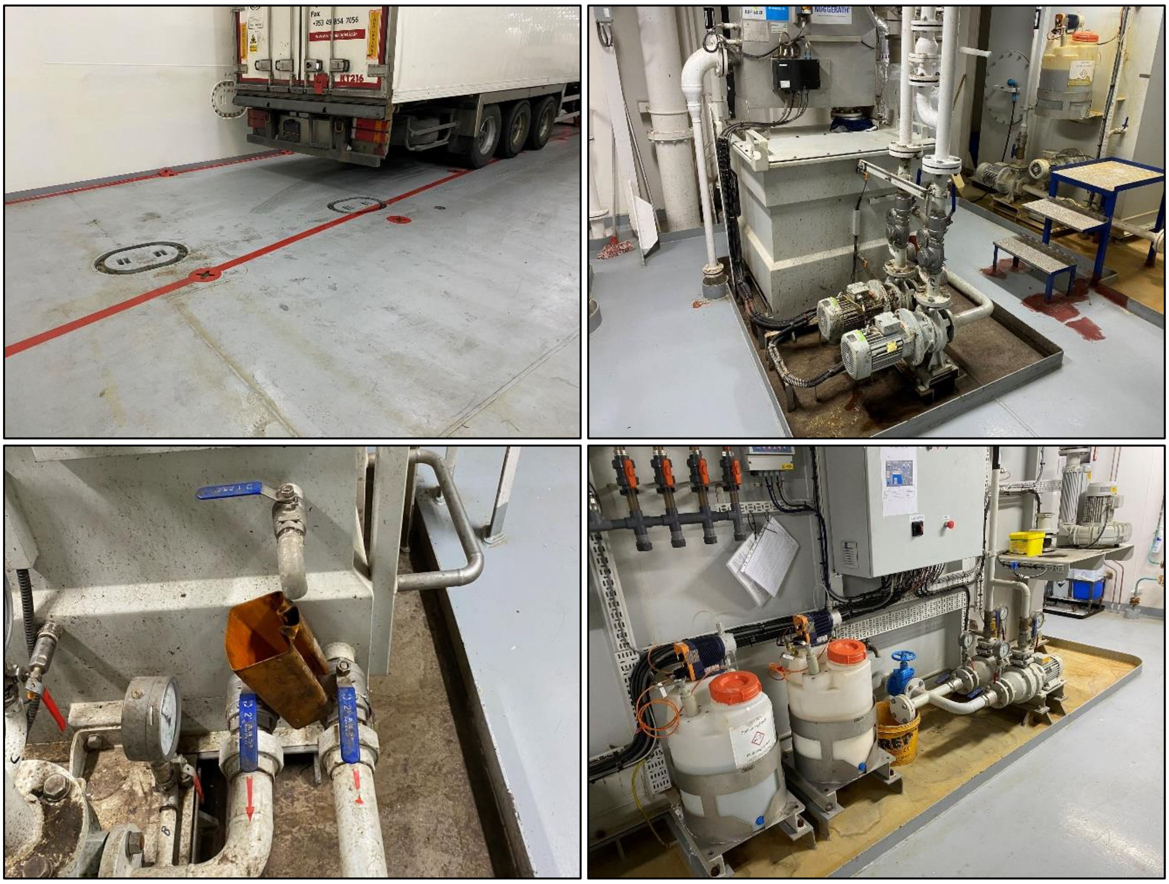

Supplementary Figure 3. Photographs from the Stena Estrid showing (i, top left) the first inaccessible blackwater holding tank located under the freight deck (note circular manholes), (ii, top right) the screening tank where samples were taken, (iii, bottom left) the sample tap for wastewater recovery, and (iv, bottom right) the on-board aerobic wastewater treatment plant.

Supplementary Table 1. Summary of the timetable for the two ships evaluated in this study.

Ship	Outward journey		Return journey	
	Holyhead departure time	Dublin arrival time	Dublin departure time	Holyhead arrival time
Stena Adventurer	02:15	05:30	08:15	11:50
	14.45	18.00	20:30	00:01
Stena Estrid	09.00	12.15	02:15	05:45
	20.30	23.45	14:45	18:20

Supplementary Table2. Summary of the (RT-)qPCR primers and probes and limits of detection and quantification (LOD and LOQ) values in genome copies (gc) per µl wastewater-derived nucleic acid extracts for the target viruses. *Fluorophores were modified to be compatible with QuantStudio 6 environment.

Assay type	Target virus	Target region (reference)	qPCR standards	Oligo type	Sequence and concentration/reaction	pmol in reaction mix	LOD (gc µl^{-1})	LOQ (gc µl^{-1})
Duplex RT-qPCR	SARS-CoV-2	N1 gene fragment (CDC, 2020)	In-house ssRNA standard	Forward primers	GACCCCAAAATCAGCGAAAT	10	0.92	12.60
				Reverse primers	TCTGGTTACTGCCAGTTGAATCTG	20		
				Probe	**FAM**-ACCCCGCATTACGTTTGGTGGACC-**MGB**	2.5		
	Phi 6 phage	phi-6S_1 gene, coding P8 protein (Gendron et al., 2010)	In-house ssRNA standard	Forward primers	TGGCGGCGGTCAAGAGC	10	18.98	52.56
				Reverse primers	GGATGATTCTCCAGAAGCTGCTG	20		
				Probe	**VIC**-CGGTCGTCGCAGGTCTGACACTCGC-**QSY***	2.5		
Duplex RT-qPCR	Influenza A	Matrix protein gene (Shu et al., 2021)	Synthetic influenza H1N1 (2009) RNA control (Twist Bioscience, USA)	Forward primers	CAAGACCAATCYTGTCACCTCTGAC<br>CAAGACCAATYCTGTCACCTYTGAC	10	1.83	7.12
				Reverse primers	GCATTYTGGACAAAVCGTCTACG<br>GCATTTTGGATAAAGCGTCTACG	20		
				Probe	**FAM**-TGCAGTCCT/**ZEN**/CGCTCACTGGGCACG-**IABkFQ**	5		
	Influenza B	Non-structural protein gene (Shu et al., 2021)	Synthetic influenza B RNA control (Twist Bioscience, USA)	Forward primer	TCCTCAAYTCACTCTTCGAGCG	10	1.18	4.60
				Reverse primer	CGGTGCTCTTGACCAAATTGG	20		
				Probe	**YakYel**-CCAATTCGA/**ZEN**/GCAGCTGAAACTGCGGTG- **IABkFQ**	5		
Triplex RT-qPCR	Norovirus GII	RdRp-ORF1 (ISO, 2013)	Plasmid DNA	Forward primer	ATGTTCAGRTGGATGAGRTTCTCWGA	10	1.60	9.20
				Reverse primer	TCGACGCCATCTTCATTCACA	20		
				Probe	**DO**-AGCACGTGGGAGGGCGATCG-**DDQ***	5		
	Entero-viruses	Untranslated region (Public Health Wales)	Synthetic enterovirus D68 RNA control (Twist Bioscience, USA)	Forward primer	GGTYGAAGAGYCTATTGAGC	10	6.25	50.00
				Reverse primer	GCTCCGYIGTTRGGATTAGCCG	20		
				Probe	**HEX**-TCCTCCGGCCCCTG-**BHQ***	5		
	Enterovirus D68	ORF1–ORF2 junction region (Poelman et al., 2015)	Synthetic enterovirus D68 RNA control (Twist Bioscience, USA)	Forward primer	TGTTCCCACGGTTGAAAACAA	10	3.10	12.50
				Reverse primer	TGTCTAGCGTCTCATGGTTTTCAC	20		
				Probes	**FAM**-TCCGCTATAGTACTTCG-**MGB**<br>**FAM**-ACCGCTATAGTACTTCG-**MGB**	5		
